# How equitable are GP practice prescribing rates for statins?: an ecological study in four primary care trusts in North West England

**DOI:** 10.1186/1475-9276-6-2

**Published:** 2007-03-27

**Authors:** Paul R Ward, Peter R Noyce, Antony S St Leger

**Affiliations:** 1 Department of Public Health, Flinders University, Adelaide, Australia; 2 School of Pharmacy and Pharmaceutical Sciences, University of Manchester, UK; 3 School of Epidemiology and Health Sciences, University of Manchester, UK

## Abstract

**Background:**

There is a growing body of literature highlighting inequities in GP practice prescribing rates for a number of drug therapies. The small amount of research on statin prescribing has either focussed on variations rather than equity *per se*, been based on populations other than GP practices or has used cost-based prescribing rates.

**Aim:**

To explore the equity of GP practice prescribing rates for statins, using the theoretical framework of equity of treatment (also known as horizontal equity or comparative need).

**Methods:**

The study involved a cross-sectional secondary analysis in four primary care trusts (PCTs 1–4) in the North West of England, including 132 GP practices. Prescribing rates and health care needs indicators (HCNIs) were developed for all GP practices.

**Results:**

Scatter-plots revealed large differences between individual GP practices, both within and between PCTs, in terms of the relationship between statin prescribing and healthcare need. In addition, there were large differences between GP practices in terms of the relationship between actual and expected prescribing rates for statins. Multiple regression analyses explained almost 30% of the variation in prescribing rates in the combined dataset, 25% in PCT1, 31% in PCT3, 51% in PC4 and 58% in PCT2. There were positive associations with variables relating to CHD hospital diagnoses and procedures and negative associations with variables relating to ethnicity, material deprivation, the proportion of patients aged over 75 years and single-handed GP practices.

**Conclusion:**

Overall, this study found inequitable relationships between actual and expected prescribing rates, and possible inequities in statin prescribing rates on the basis of ethnicity, deprivation, single-handed practices and the proportion of patients aged over 75 years.

## Background

The authors of this paper published a recent paper in this journal which attempted to explain the variations in GP prescribing rates for a range of coronary heart disease (CHD) drugs [1]. The findings were based on multiple regression analyses, whereby the dependent variables were prescribing rates for different CHD drugs, and the independent variables were specifically developed proxies of healthcare need (e.g. CHD mortality and morbidity rates, demographics of the patient population, socio-economic status of the patient population etc). The paper showed that variations in GP prescribing rates could be explained to vastly differing degrees and were also related to different proxies of healthcare need. For example, there were negative relationships between prescribing rates and patient age, deprivation and ethnicity. From these findings, and other related papers published by the authors [2-5], we concluded that GP prescribing rates were inequitable. The present paper uses data from the same study to further explore the issue of equity of prescribing. This time, we specifically examine the equity of statin prescribing using a variety of descriptive, visual and multivariate analyses. Our focus on statin prescribing stems from the huge international interest in the prescribing of drugs to reduce cholesterol and the associated problems of prescribing statins to all those people who 'need' them. In other words, prescribing of statins represent an important case-study on which to situate an exploration of equity.

### Aims of the paper

The overall aim of this paper is to provide further evidence on the equity of GP practice prescribing rates for statins across and within 4 primary care trusts (PCTs) in the North West of England. This is mainly achieved by exploring the associations between statin prescribing rates and proxies of health care need (called health care needs indicators (HCNIs) throughout this paper), although we also explore the associations between actual and expected prescribing rates for statins.

### Importance of statins in the management of CHD

Statins are a group of drugs which are widely used in the primary and secondary prevention of coronary heart disease (CHD), and their clinical effectiveness is well known [6-9]. There has been an increasing amount of evidence about the effectiveness of statins since the mid 1990s, which has lead to increased pressures on general medical practitioners (GPs) to prescribe these drugs, although the financial implications of prescribing statins to all eligible patients may mean that such a strategy is not affordable [10]. This conundrum lead one commentator to pose the question, "*How can GPs prescribe in line with the evidence yet manage to remain within budget?*" [11], and for others to suggest that statin prescribing should be prioritised for those patients with the highest levels of health care need [12]. Therefore, since statins cannot be prescribed to all patients with clinical need (due to financial considerations), research around the equity of statin prescribing is extremely important and timely.

Since publication of the trials on the effectiveness of statins, there has been an overall increase in the level of statin prescribing within the UK, although this has not occurred to the same extent across GP practices [13-15] or across all patient groups with established coronary heart disease within GP practices [16,17]. Whilst we recognise that variations in statin prescribing exist, less consensus exists on how to quantify and understand the equity of such variations. The need to understand the nature and cause of such variation remains paramount since consistency of service for its own sake may be pointless [18].

Studies attempting to explain the variation in statin prescribing rates have been modest, with most studies explaining around 20 per cent of the variation [14,16,19,20]. The prevalence of CHD explained 12 per cent of the variation in statin prescribing in men, and 7 per cent in women [16], deprivation explained 14 per cent [14], and a combination of nitrate prescribing rates and population aged between 35 and 74 years explained 18 per cent [20]. Therefore, the majority of variations in statin prescribing rates remain unexplained.

### Importance of researching the equity of GP prescribing

One of the most important principles of health care systems in the developed world is based around the notion of equity. Within the UK, the National Health Service (NHS) was set up to provide a universal entitlement to the same quality of health care services solely on the basis of clinical need [21,22]. There are large literatures around how to define, operationalise and measure equity in relation to health care services [22-24], although equity is generally taken to mean 'fair' or 'just'. Equity has been divided into three domains: equal *access *to health care for people in equal need; equal *treatment *for people in equal need; and equal *outcomes *for people in equal need [22]. Whilst this is a simplification of the nature of equity, it is useful in delineating the various domains in which inequities may arise.

The current paper is focussed around the equal treatment for people in equal need (i.e. horizontal equity), since variations in prescribing may arise from the interaction between supply and demand which depend on a number of factors relevant to both patients and GPs (recognition of symptoms, knowledge of services, preferences for treatment, GP-patient interaction etc). This notion of equity is also akin to comparative need, which is one domain in the taxonomy of need [25,26]. Comparative need is determined by studying the characteristics of differing populations in receipt of differing levels of a service (e.g. differing rates of prescribing). Using the example of the current study, a comparative approach to need would assess the differences in prescribing rates provided to the population of one GP practice compared to another GP practice, weighted to take account of the relevant risk factors in their patient populations. However, it needs to be remembered that this approach is purely comparative. Therefore, if the population of GP practice A is deemed to be in need in comparison to the population of GP practice B, this does not necessarily mean that the population of GP practice B is not in need – the prescribing rates of GP practice B may not be at an adequate level. This approach merely attempts to assess comparative need (or equity), and makes no judgements about the appropriateness of prescribing.

The concept of equity of prescribing is extremely important in the area of prescribing, since it informs us of the groups of patients who are currently receiving these drug therapies (and maybe do not need the drugs) and those who are currently not receiving these drug therapies (and maybe do need the drugs). There is a sound evidence base in terms of the effectiveness and which patients may benefit from statins [6,9,27-29] although our evidence base in terms of who actually receives the drugs in practice is less well developed. Obviously, it is also crucial to understand which patients also benefit from the drugs (equity of outcomes), although that is not within the remit of this paper. This paper is aimed at the interface between who could benefit from these drugs (eg older populations, South Asian populations, deprived populations, populations with a high prevalence of CHD and/or a high mortality rates from CHD), and who actually receives the drugs.

### Evidence on the equity of statin prescribing

Although there is overwhelming evidence as to the effectiveness of statins, there seems to be a "*treatment gap*" [30] between those people for whom treatment is indicated, and those people who actually receive it. Indeed, at a conference held by the Royal College of Physicians in Edinburgh, it was suggested that although the use of statins has increased substantially in Great Britain over the last few years, "*many people who would benefit from the drugs are still not receiving them*" [31].

A number of studies have explored the treatment gap for statins at the individual level, although these have not explored the equity of prescribing *per se*. Some UK studies have found that only around 20 to 30 per cent of people with CHD are prescribed statins [32], although studies in the US and Europe have found higher prevalences (around 40 per cent) of statin use in patients with CHD [33-37]. Sub-optimal treatment has also been found for patients with cholesterol concentrations above recognised treatment limits. In two UK based studies, the majority of people with a history of CHD were not taking a statin, but had cholesterol levels above that at which treatment with statins is recommended [32,38]. In addition, over half those who were taking statins still had high cholesterol levels, possibly due to the low doses prescribed.

Whilst a number of studies have highlighted variations in the access to, and provision of CHD hospital interventions on the basis of patient age, gender, ethnicity and socio-economic status, [39-43] there is only a small, but growing, literature which has focussed on the equity of statin prescribing rates at a population level. Statin prescribing has been shown to vary between health authorities, PCTs and GPs [4,5,13-16] and between patients on the basis of gender, age and ethnicity [4,16,19,43-46]. Prescribing rates of statins are positively associated with GP diagnoses of CHD [16] and with expected rates of CHD prevalence [4,19]. However, characteristics of GP practices such as their training status, the number of GPs, or their single-handed status have been found to have no relationship with prescribing rates for statins [14,20].

## Methods

This section focuses on the setting for the study and the data sources and methods used to develop actual and expected prescribing rates and proxies for health care need (called health care needs indicators (HCNIs) in this paper), which were developed for each GP practice in the 4 PCTs. More details of the data sources and methods used can be found in our previous paper [1] and elsewhere [4,5]. Local Research Ethics Committee approval was granted for this study.

### Setting

The cross-sectional, ecological study was undertaken in four primary care trusts (PCTs) in the North West of England (called PCT1, PCT2, PCT3, and PCT4 throughout this paper). GP practices with fewer than 1000 registered patients were excluded from analysis (n = 16). After excluding these GP practices, there were 132 GP practices (50 in PCT1, 24 in PCT2, 31 in PCT3, and 27 in PCT4) with a combined registered population of over 350,000 patients aged over 35 years.

### Developing actual prescribing rates for statins

When an NHS prescription is dispensed in primary care, the prescription form is sent to the Prescription Pricing Authority for processing, which collates these data and provides them to GP practices and PCTs in the form of Prescribing Analysis and Cost (PACT) data. PACT data are available for all GP practices in England, and allow detailed interrogation in terms of drugs prescribed along with their dosages, pack sizes and formulations. For example, for a specific time period, we can collect data on which statins were prescribed by a GP practice in addition to the dosages and pack sizes. This allows for a complex and timely analysis of PACT data. Useful critiques of PACT data can be found elsewhere [47,48].

Prescribing analysis and cost (PACT) data were obtained for all GP practices in the 4 PCTs for the 12-month period October 1999 to September 2000. At the time of data collection, there were five statins in the BNF, and therefore available to prescribe in primary care. These statins were Atorvastatin, Cerivastatin, Fluvastatin, Pravastatin, and Simvastatin, and PACT data were collected for all of them. However, Cerivastatin was withdrawn from use in 2001. Nevertheless, since Cerivastatin was licensed for use during the period of data collection, it remains part of the PACT data used in subsequent analysis. The prescribing rate was calculated for all statins combined.

The denominator for the prescribing rate was the total registered (and resident) patient population aged over 35 years. This age group was chosen since the prevalence of CHD is particularly low in people aged less than 35 years [49]. The numerator was Average Daily Quantities, which are discussed in more detail in our previous paper [1] and elsewhere [50-52].

### Developing expected prescribing rates for statins

Data were collected in the General Practice Research Database on age-sex rates of prescribing for statins, which are presented in Table 1.

**Table 1:** Age-sex prescribing rates for specific CHD drug groups

**Sex**	**35–44**	**45–54**	**55–64**	**65–74**	**75–84**	**85+**
**Male**	6.1	26.0	56.7	67.5	25.5	2.6
**Female**	2.0	10.4	38.5	57.0	21.6	2.4

Source: [62]

The General Practice Research Database derives data from a representative sample of GP practices in England (211 GP practices with a combined population of around 1.4 million patients) about the NHS care received by their registered patients [49]. This group of GP practices regularly collects data for the General Practice Research Database. From these data, expected prescribing rates of statin prescribing were calculated by applying the age-sex specific rates to the same age-sex groups within the registered populations of all GP practices in the study. In this way, an expected rate of statin prescribing was calculated, based on the demographics of the GP practice registered population. It should be remembered that these prescribing rates are not the 'clinically correct' prescribing rates, but a nationally representative sample on which to compare GP prescribing within our study sample.

### Developing health care needs indicators (HCNIs)

Again, the data sources and methods used to develop the HCNIs are discussed in much more detail in our previous paper [1]. However, we will briefly outline them here in order for the reader to be able to interpret the findings from this paper.

In total, 24 HCNIs were developed for each GP practice in this study, and all of these were entered into multiple regression models. A list of the HCNIs developed are provided in Appendix A, although the only HCNIs discussed here are those included in the final regression models (i.e. those HCNIs which had independent, statistically significant associations with statin prescribing rates). It is recognised that all of the variables discussed here do not relate specifically to health care need (some relate to supply and others to administrative factors), although the term is used here to identify the set of variables used in the analyses.

Demographic HCNIs were developed directly from GP practice list data, and these relate to the percentage of patients aged 55–74 years, and the percentage aged over 75 years. Both of these demographic groups are indicators of health care need for CHD drugs [49]. The Low Income Scheme Index was used as a proxy for low income since it represents the percentage of prescriptions which are exempt from prescription charges due to low income [53]. The percentage of patients from all ethnic minority groups was estimated for each GP practice using data from the 1991 census. The method of patient weighted attribution was used to develop these estimates using data at enumeration district level [54-56].

Data were also obtained from hospital episode statistics on specific hospital procedures (coronary artery bypass graft (CABG), percutaneous transluminal angioplasty (PTCA) and coronary angiogram) and diagnoses (primary diagnosis of CHD) [57]. Although hospital episode statistics relate to the supply, as opposed to need for health care services, it was hypothesised that in the absence of other CHD morbidity data, they may represent a useful proxy of CHD morbidity in GP practice populations. Crude rates (per 1000 patients aged over 35 years) were calculated for CHD procedures (CABGs, PTCAs and angiograms) and CHD diagnoses. In addition, overall rates of CHD hospital episodes were calculated (diagnoses + procedures) which are called CHD hospital episode statistics throughout this paper.

Two additional variables were examined: number of full-time equivalent GPs per 1000 registered patients and whether the practice was single-handed. Whilst these variables do not relate specifically to health care need, they have been shown to affect service provision and the equity of health care, and therefore were included in analyses.

### Data analysis

Correlations between actual and expected prescribing rates were calculated using Spearman's Rank Correlation Coefficient and the Mann-Whitney test was used to detect differences in mean prescribing rates between single-handed and multiple partner practices. Multiple linear regression modelling was undertaken for in each PCT in addition to the combined dataset (all data were normally distributed). Analysis was undertaken within both PCTs and the overall dataset in order to further understand differences between PCTs – by solely focusing on the combined dataset, we would ignore any important differences between PCTs.

The dependent variable in each model was the statin prescribing rate and the independent variables were the HCNIs developed in the study. The forward-stepwise approach was taken and the final models were checked for collinearity and normality of residuals. The final regression models only contained the HCNIs which had statistically significant (p < 0.05) independent associations with the dependent variable, and each model was checked for collinearity and normality of residuals. Overall, for each multiple regression model, all 24 HCNIs were entered as independent variables, and the final model included only those variables that were statistically significant and added to the fit of the model.

## Results

**Figure 1:**
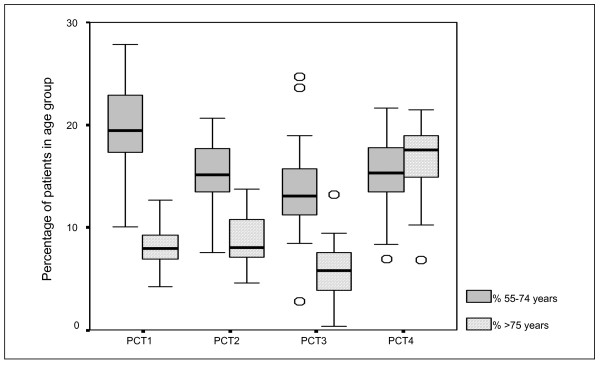
Box-plot of patient demographics by PCT.

**Figure 2:**
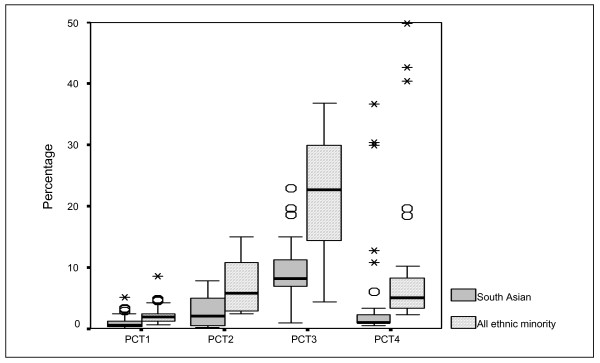
Box-plot of ethnicity by PCT.

**Figure 3:**
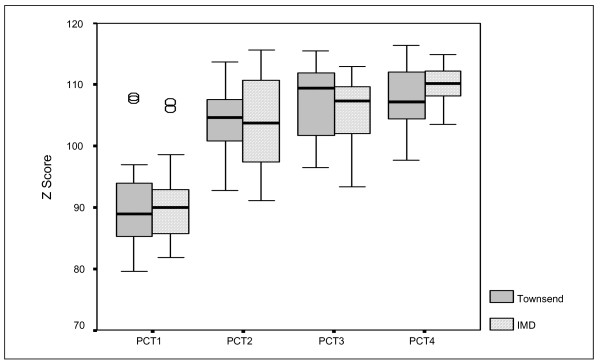
Box-plot of multiple deprivation by PCT.

**Figure 4:**
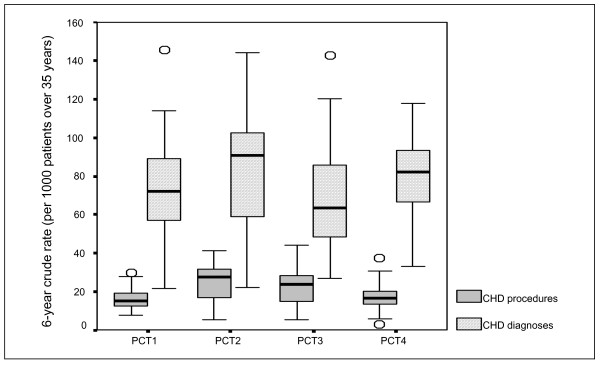
Box-plot of hospital procedures and diagnoses by PCT.

Details about the 'health needs' of populations of the PCTs are provided in Figures 1, 2, 3, 4. These represent the demographics (Figure 1), ethnicity (Figure 2), multiple deprivation (Figure 3) and CHD hospital interventions (Figure 4). A general overview of comparative health care need suggests that PCT4 had the highest levels of CHD related health care needs within the study, whereas PCT1 had the lowest health care needs. PCT4 was the most deprived of all PCTs and had the highest proportions of patients aged over 75 years. In contrast, PCT1 may be seen as the 'least needy' of all PCTs on the basis of the HCNIs developed in this study. PCT1 was the least deprived, had the lowest proportions of South Asian groups and the lowest median rate of CHD hospital procedures. However, PCT1 had the highest median percentage of patients aged between 55 and 74 years which may well be the target age-group for prescribing within CHD.

### Associations between actual and expected prescribing rates

There were statistically significant correlations (p < 0.05) between actual and expected prescribing rates in PCT2 (r = 0.407), PCT3 (r = 0.366), PCT4 (r = 0.43) and the combined dataset (r = 0.438). Although statistically significant, the magnitude of associations were rather low, suggesting that actual prescribing rates are not highly associated with expected prescribing rates based on the data in the General Practice Research Database. Indeed, in PCT1, the correlation was non-significant. Whilst this does not infer inequitable prescribing, it does suggest that prescribing patterns in these PCTs does not conform to what we may expect given their age-sex compositions.

**Figure 5:**
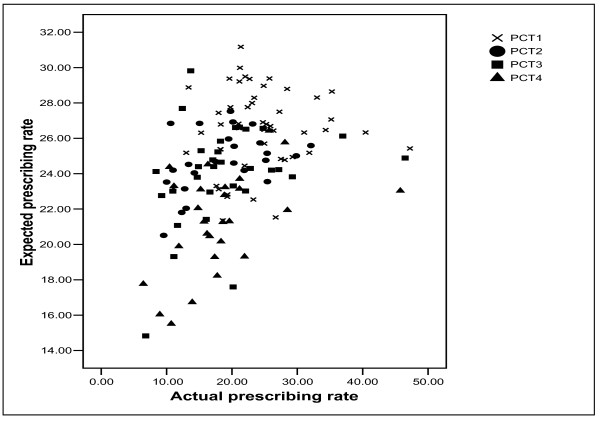
Scatter-plot of actual and expected statin prescribing rates.

Figure 5 presents a scatter-plot to show the variation between the 132 GP practices and PCTs in terms of the association between actual and expected prescribing rates. The generally positive association in the combined dataset can be seen in the scatter-plot, although the large degree of variation between individual GP practices is also apparent. For example, in PCT3, one GP practice has the lowest expected prescribing rate and another has the third highest, although their actual prescribing rates are fairly similar. If one draws a vertical line on the scatter-plot with an actual prescribing rate of 15 Average Daily Quantities per patient aged over 35 years, there is around a two-fold difference in the expected prescribing rates between the GP practices.

### Associations between statin prescribing rates and HCNIs

The multiple regression models for each PCT in addition to the combined dataset are presented in Table 2, reveals that between 25% and 58% of the variation in prescribing rates could be explained between the individual PCTs. When regression analysis was undertaken for the combined dataset, we could explain almost 30% of the variation.

**Table 2:** Multiple regression models

**PCT**	**R**^**2**^	**Needs indicator**	**Standardised beta coefficient**	**% variance explained**
PCT1 (*n *= 50)*	.245	CHD diagnoses rate	.489	15.0
		WTE GPs/1000 patients	-.327	9.5
PCT2 (*n *= 24)*	.583	CHD hospital episode statistics rate	.763	58.3
PCT3 (*n *= 31)*	.313	CHD procedures rate	.490	20.7
		Low Income Scheme Index	-.327	10.6
PCT4 (*n *= 27)*	.505	Single handed	-.340	27.0
		Ethnicity	-.538	12.5
		% patients aged >75	-.373	11.0
Combined (*n *= 132)*	.289	CHD hospital episode statistics rate	.350	14.7
		% patients aged >75	-.240	4.2
		Ethnicity	-.233	3.1
		% patients aged 55–74	.199	2.9

* *n *refers to the number of GP practices in each PCT which were contained in the regression model

**Figure 6:**
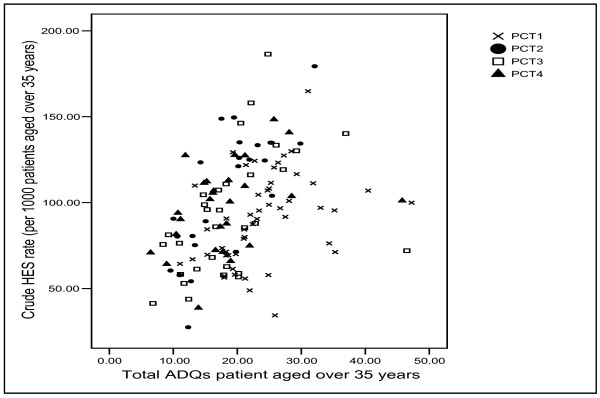
Scatter-plot of statin prescribing rates and CHD HES rate.

One of the most important findings here was that hospital episode statistics were strongly (and positively) related to prescribing rates in three PCTs and in the combined dataset. The overall CHD hospital episode statistics rate explained 58% of the variation in prescribing in PCT2, the rate of CHD diagnoses explained 15% of the variation in PCT1 and the rate of CHD procedures explained 21% of the variation in PCT3. Figure 6 shows a scatter-plot of prescribing rates against the CHD hospital episode statistics. This reveals the generally positive association between statin prescribing rates and the CHD hospital episode statistics rate, and also the difference between the pattern for PCT2 and the other PCTs. The scatter-plot also reveals the large differences between GP practices within the same PCT. For example, in PCT3, there are a number of GP practices with prescribing rates around 20–25 Average Daily Quantities per patient aged over 35 years, although there is around a 3-fold difference in terms of CHD hospital episode statistics. This pattern is suggestive of inequities in prescribing rates.

In PCT4, all three variables had negative beta coefficients, suggesting that single-handed practices and those with higher proportions of ethnic minority patients and patients aged over 75 years were more likely to have lower prescribing rates. The mean prescribing rate in single-handed practices throughout the combined dataset was 18.6 (SD 9.5) Average Daily Quantities per patient aged over 35 years and the corresponding rate for multiple partner practices was 20.7 (SD 7.1). This difference was statistically significant (p = 0.042) using the Mann Whitney test.

**Figure 7:**
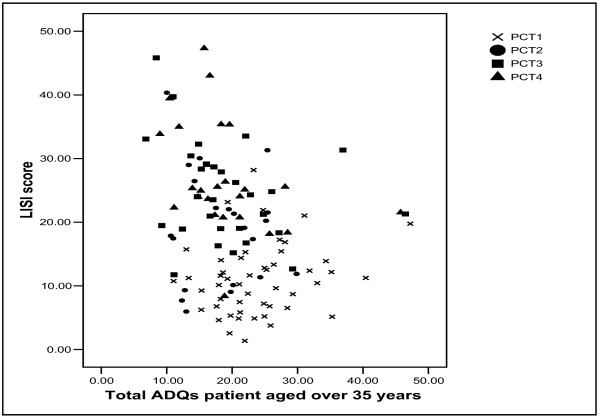
Scatter-plot of statin prescribing and LISI score.

The Low Income Scheme Index score explained 11% of the variation in prescribing rates in PCT3, and the beta coefficient was negative, suggesting that GP practices with higher Low Income Scheme Index scores had lower prescribing rates. Figure 7 shows a scatter-plot of prescribing rates and the Low Income Scheme Index score. This is suggestive of a negative association in PCTs 2 to 4, and a lack of association in PCT1. Again, this scatter-plot reveals large difference within PCTs. For example, in PCT4, there are a number of GP practices with prescribing rates around 10 to 20 Average Daily Quantities per patient aged over 35 years, although their Low Income Scheme Index scores almost 4-fold. Again, this is suggestive of inequities in prescribing rates.

## Discussion

The regression models explained more variation in prescribing rates than those found in other studies. For example, most studies have explained around 20 per cent of the variation [14,16,19,20], whereas 30 per cent of the variation was explained in the combined dataset. In addition, analyses for individual PCTs revealed that 50 per cent of the variation in PCT4 and almost 60 per cent in PCT2 could be explained.

The HCNIs developed from hospital episode statistics were present in the regression models for three PCTs, and were positively related to prescribing rates. Another study [58] also used hospital episode statistics for primary diagnoses of CHD as a proxy for health care need, although the authors did not find a positive relationship between rates of statin prescribing and CHD hospital episode statistics. Nevertheless, other studies have also found positive relationships between rates of PTCAs and statin prescribing [35,38,59].

The percentage of patients aged 55–74 years was generally positively related to prescribing rates, suggesting that prescribing rates are related to health care need. Whilst this HCNI only explained 3 per cent of the variation in prescribing rates, a number of studies have found that statin prescribing is higher in this age group, than in older age groups [16,32,36,59], with one study finding that the proportion of patients aged 35–74 years explained 5 per cent of the variation in statin prescribing rates between GP practices [20]. The percentage of patients aged over 75 years had a negative relationship with prescribing rates in PCT4 and the combined dataset. A number of studies found that older patients were much less likely than younger patients to receive a prescription for a statin [16,32,36,59], which may result from the lack of research evidence on the efficacy of statins in elderly populations. Therefore, although the percentage of patients aged over 75 years is a proxy for CHD prevalence, it may not represent a useful proxy of the potential to benefit from statins.

The findings from this study provide conflicting evidence as to the relationship between prescribing rates and deprivation. In PCT1, PCT2 and PCT4, there was little evidence of associations between deprivation and prescribing rates, although statin prescribing rates in PCT3 were associated with the Low Income Scheme Index score (negative association). Therefore, there does not seem to be a simple relationship between prescribing rates and deprivation which applies in all PCTs. Since CHD prevalence increases in deprived populations, one would expect a positive association between prescribing rates and the Low Income Scheme Index score, and therefore the negative association in PCT3 and lack of association in the other PCTs is suggestive of inequities in prescribing rates.

The estimated proportion of patients from ethnic minority groups was negatively associated with prescribing rates in PCT4 and the combined dataset, which is similar to findings elsewhere [44,46]. However, the construction of the ethnic minority HCNI in this study was based on enumeration district populations, often with low numbers of ethnic minority populations, and therefore further work would be required to provide a rigorous assessment of the suggestion being made in this study. Nevertheless, these patterns add further weight to suggestions about the inequity of prescribing rates on the basis of ethnicity.

Although the HCNIs developed from GMS data do not necessarily reflect health care needs, they were nevertheless important in explaining variations in prescribing rates. In general, single-handed GP practices had lower prescribing rates than multiple partner GP practices and the number of WTE GPs per 1000 patients had negative associations with rates of statin prescribing PCT1. A number of studies have found that organisational factors such as the ones used in this study were not associated with statin prescribing rates [14,20]. However, the importance and impact of health care supply has been recognised in the recent allocation formula for prescribing budgets, which includes variables related to training and single-handed GP practices in addition to the number of GPs per patient [60]. Indeed, in developing the allocation formula, the authors also found that prescribing rates were negatively associated with single-handed GP practices.

### Main strengths of the paper

Firstly, the study explicitly focussed on the equity of prescribing, as opposed to solely explaining the variation in prescribing rates. In this way, this paper extends our previous paper in this journal. Secondly, the study was based on all GP practices (with practice lists over 1000 patients aged over 35 years) in 4 PCTs, rather than a random sample such as those in the General Practice Research Database. Therefore, the results are directly applicable to the PCTs and enable them to develop local policies/guidance without the need to infer from GP practices in other areas. Thirdly, we used prescribing rates based on Average Daily Quantities, which are more applicable to UK general practice, and also based on the population at risk from CHD (those aged over 35 years). As already stated, international comparisons may wish to use Defined Daily Doses. Fourthly, we used a wide range of data sources for the HCNIs, rather than relying solely on the 1991 Census. Fifthly, we developed expected prescribing rates for all GP practices which may be used by the PCTs to audit changes in the equity of prescribing rates over time. Finally, we produced scatter-plots which enable the identification of individual GP practices which seem to either have higher or lower actual than expected prescribing rates. Further work could then be undertaken within these GP practices in order to understand the reasons behind their apparent inequitable prescribing rates and subsequently to provide education and support to make their prescribing rates more equitable.

### Main weaknesses of the paper

Firstly, ecological analyses cannot be used to infer causal relationships or to infer similar relationships at an individual level (the ecological fallacy). Future studies may take an approach based on multi-level analysis, although this was outside the scope of this study. Secondly, the study size was determined by considerations of practicability rather than study power. Thus although the positive findings remain valid and interesting it is possible that true, but lower magnitude, relationships exist between Statin prescription and some of the variables that dropped out of our regression models. Thirdly, PACT data refer only to NHS scripts that have been dispensed at pharmacies, rather than all prescriptions issued by GPs. Thirdly, hospital episode statistics data can be limited by lack of accuracy and completeness and only refers to diagnoses and procedures in NHS hospitals. Finally, the data on statin prescribing rates is based on 1999–2000 data, which may be slightly out of date given the recent increases in statin prescribing. However, we have no evidence that this increase has increased the equity of prescribing rates, since it may have increased at a similar rate across all GP practices. Nevertheless, it may be useful to provide data for 2005–2006 to confirm or reject this.

## Conclusion

Overall, this study has found that statin prescribing rates may be explained (to differing degrees between PCTs) by a mixture of HCNIs relating to both health care need and supply. Rates of CHD procedures and diagnoses were generally positively associated with prescribing rates, although the percentage of ethnic minority patients, the percentage of older (>75 years) patients, deprivation and GP administrative factors were negatively associated in some PCTs. Therefore, the findings from this study show that GP practice prescribing rates for statins are generally inequitable, although the strength of this inequity and the patient groups involved (ethnic minority, older and deprived patients) differ between PCTs.

The National Service Framework for coronary heart disease [61] has highlighted variations in the quality and access to CHD services in the UK. However, the equity of CHD services such as GP prescribing rates has been a neglected area of research. This study adds to weight to the assertions of the National Service Framework about the inequitable supply of CHD services and may form the baseline for further studies to assess the effectiveness of the National Service Framework in reducing the inequities in prescribing rates.

## Competing interests

The author(s) declare that they have no competing interests.

## Authors' contributions

PW was awarded funding for this study, was involved in the conception, design and managed the day to day running of the study, undertook all data collection and analysis, and wrote the paper. PN and ASL were involved in the conception and design of the study, made active contributions in project meetings, were involved in an advisory capacity in all aspects of the project and advised on drafts of the paper. PW is the guarantor.

## Appendix A – List of health care needs indicators (HCNIs) developed during the study

• Proportion of patients aged between 55 and 74 years

• Proportion of patents aged over 75 years

• Proportion households with no car

• Proportion males who are economically inactive

• Townsend Score

• Proportion of households receiving council tax benefits

• Proportion unemployment

• Index of Multiple Deprivation

• Income Deprivation Index

• Low Income Scheme Index (LISI) score

• Standardise mortality rate (SMR) for CHD under 75 years

• 6-year crude rate of coronary artery bypass grafts (CABGs) per 1000 patients

• 6-year crude rate of percutanious transluminal angioplasty (PTCAs) per 1000 patients

• 6-year crude rate of coronary angiograms per 1000 patients

• 6-year crude rate of CHD hospital procedures (CABGs + PTCAs + angiograms) per 1000 patients

• 6-year crude rate of CHD hospital diagnoses per 1000 patients

• 6-year crude rate of CHD prevalence (diagnoses + procedures) per 1000 patients

• Regionally specific prevalence, age and sex standardised prescribing units per patient over 35 years (PASS-PUs)

• Proportion of population defining themselves as 'non-white'

• Proportion of population defining themselves as 'South Asian'

• Proportion of population over 30 with a limiting long-term illness (LLI)

• Health Deprivation Index

• Proportion of households with more than 2 cars

• Access Deprivation Index
